# Comparison of Mental Illness Comorbidity Pre-Pandemic vs. Pandemic-Era and Associations with Clinical and Demographic Characteristics for Virginia Public Hospital Inpatient Discharges with a Substance Use Disorder

**DOI:** 10.3390/ijerph23010129

**Published:** 2026-01-21

**Authors:** Marilyn Bartholmae, Tharidu Gunawardena

**Affiliations:** 1 Department of Psychology, Liberty University, Lynchburg, VA 24515, USA; 2 Macon & Joan Virginia Health Sciences, Eastern Virginia Medical School, Old Dominion University, Norfolk, VA 23529, USA; gunawat@odu.edu

**Keywords:** substance use disorder, mental illness comorbidity, public hospitals

## Abstract

**Highlights:**

**Public health relevance—How does this work relate to a public health issue?**

- Substance use disorders are of great public health concern given their high prevalence and associated mortality, morbidity, and socioeconomic burden.
- Patient discharge data enable the study of public health issues through large population-based datasets.

**Public health significance—Why is this work of significance to public health?**

- While increases in mental illness rates are well documented, it is not known whether the increases in rates of mental illness are increases in single mental illness diagnoses or comorbid mental illness diagnoses.
- The specific associations between COVID-19, physical illness, and mental illness comorbidity have not been studied before among inpatients with SUD.

**Public health implications—What are the key implications or messages for practitioners, policy makers, and/or researchers in public health?**

- It is important for healthcare leaders to be aware of increases in comorbid mental illnesses as they may interact and affect the course and prognosis of these illnesses.
- These findings are important for healthcare leaders as new highly transmissible coronavirus subvariants are rising worldwide, chronic physical illnesses such as obesity and hypertension remain major healthcare challenges, and SUD and mental illness rates continue to increase.

**Abstract:**

The rise in mental illnesses after the COVID-19 pandemic is well documented. However, it is not known whether the rates of mental illness comorbidity increased. The objectives of this study were to compare mental illness comorbidity rates before and after the pandemic among inpatients with SUD and to test associations between mental illness comorbidity, physical illness, and demographics. We used a retrospective cross-sectional design in a sample of inpatient discharges (N = 233,017) at Virginia public hospitals from January 2018 to December 2022. We used Z tests to compare rates of mental illness comorbidity pre- and post-pandemic and Chi-square tests to examine associations of mental illness comorbidity with physical illness and demographics. Single and comorbid mental illness significantly increased from pre- to post-pandemic, *p* < 0.0001. Mental illness comorbidity was significantly associated with sex, age, race, insurance, COVID-19/Long COVID, HIV/AIDS, COPD, hypertension, obesity, CVD, cancer, and diabetes (*p* < 0.0001). There was a significant increase in mental illness comorbidity, which was significantly associated with age, race, sex, and physical illnesses. Children/adolescents, females, American Indians, and individuals with HIV/AIDS had the highest rates of mental illness comorbidity. Public health action is needed to address the increase in complex medical needs among people with SUD.

## 1. Introduction

Substance use disorders (SUDs) are of great public health concern given their high prevalence and associated mortality, morbidity, and socioeconomic burden. Multiple studies indicate the COVID-19 pandemic further exacerbated SUD-related morbidity and mortality long-term [1,2,3,4,5]. Mental illness and SUD have a bi-directional relationship and often co-occur [6,7,8,9]. Mental illnesses associated with SUDs also increased significantly after the pandemic. Particularly, anxiety, depression, insomnia, attention deficit hyperactivity disorder (ADHD), posttraumatic stress disorder (PTSD), and suicide ideation increased post-pandemic [1,10,11,12,13,14].

Some studies suggest people with mental illness and/or substance use problems are more likely to experience COVID-19 or Long-COVID (while there is heterogeneity in definitions for Long-COVID, one definition reported in the literature describes Long-COVID as COVID-19 signs and symptoms persisting for more than four weeks) [9,15]. Other studies indicate people with COVID-19 or Long-COVID are more likely to experience mental illness and/or substance use challenges [1,2,16]. For example, the National Institute on Drug Abuse suggests people with SUD were 1.5 times more likely to have COVID-19 compared to people without SUD [17]. Wang et al. (2021) found that patients with a recent diagnosis of SUD were significantly at increased risk for COVID-19 [18]. Tam et al. (2023) suggest that 45.5% of study participants with Long COVID had “problematic” alcohol use and over 50% use/misuse substances [5], and Bartholmae et al. (2023) suggest people with comorbid mental illnesses (having at least two co-occurring mental illness diagnoses such as depression and anxiety) are more likely to experience a higher number of COVID-19 symptoms [19]. The rise in mental illnesses after the pandemic may be partially explained by the delay in receiving necessary healthcare services due to the purposeful reduction in non-critical and critical medical services and COVID-related fears [20,21,22,23,24,25,26]. Both physical and mental health outcomes were impacted by the reduction in healthcare services during the pandemic [27].

A bi-directional relationship between physical illness and mental illness has also been found. Individuals with a chronic medical condition are more likely to develop a mental illness and vice versa [28,29,30]. For example, COVID-19 could lead to the development of brain and mental health symptoms such as cognitive and attention deficits, anxiety, depression, psychosis, seizures, and suicidal behaviors [31]. Individuals who have diabetes, cancer, cardiovascular disease, and other physical chronic illnesses are more likely to develop a mental illness compared to individuals who do not have these chronic physical illnesses [32]. Although not fully understood, a recent study suggests that the COVID-19 pathophysiology may involve alterations in the dopamine and serotonin synthetic pathways, leading to neurotransmission dysregulation/dysfunction and subsequent mental illness [33]. Similarly, other studies suggest physical and mental health are interconnected by neural systems regulating somatic physiology and cognitive functions [28,29,30,34]. Mechanisms for the development of mental illness following COVID-19 infection may be multifactorial and may interact synergistically.

Social and economic factors have been identified as important risk factors in the development of physical and mental illness [35,36]. Racial/ethnic minorities as well as people with low socioeconomic status (SES) were over-represented among people with COVID-19. They are also at an increased risk of experiencing poorer mental health outcomes due to a multitude of underlying factors such as higher stress levels, lower immunity, and a higher prevalence of chronic medical conditions compared to non-minorities and those of higher SES [37,38,39,40]. Worsened physical health outcomes among individuals of lower SES may have activated an increase in individuals with comorbid mental illness diagnoses.

While the literature reports increased rates of mental illness after the pandemic [1,10,11,12,13,14,41], it is not known whether these increases in mental illness reflected a single mental illness diagnosis or comorbid mental illness diagnoses (having two or more co-occurring mental illnesses). Since there were rising trends in drug use and drug overdose mortality after the pandemic [1,2,3,4,5], this study focuses on mental illnesses among inpatients with SUD.

To better understand mental illness co-morbidity within the context of the pandemic, this paper sought to address two research questions for individuals with SUD:

(1) Are there increases in rates of single mental illness diagnosis or increases in rates of comorbid mental illness diagnoses among individuals with SUD after the COVID-19 pandemic? We hypothesized that there would be a significant increase in comorbid mental illness rates post-pandemic compared to pre-pandemic rates. It is important for healthcare leaders to be aware of increases in comorbid mental illnesses as they may interact and affect the course and prognosis of these illnesses [42]. Currently, studies have focused on the evaluation of the prevalence of single mental illness during the pandemic. For example, a systematic review and meta-analysis including 66 studies evaluated the prevalence of depression, anxiety, distress, and insomnia during the pandemic. Studies included cross-sectional, case-control, cohort, and intervention designs. The prevalences of these mental health problems were evaluated separately. The authors suggest increases in mental illness during the pandemic, and chronic diseases such as cancer and type 2 diabetes as risk factors for mental illness [14]. There is a clear need for research assessing the prevalence of comorbid mental illness.
(2) Is mental illness comorbidity associated with COVID-19/Long COVID, chronic physical illness, or demographic characteristics among inpatients with an SUD diagnosis? We hypothesized that mental illness comorbidity would be significantly associated with COVID-19/Long COVID, physical illness, and demographic characteristics for individuals with SUD. Findings may provide preliminary knowledge about vulnerable SUD populations who may need integrated medical and behavioral interventional approaches to alleviate the growing SUD-related morbidity and mortality rates.

## 2. Materials and Methods

### 2.1. Study Design

Using a retrospective cross-sectional design, this study examined whether mental illness comorbidity, COVID-19/Long COVID, sociodemographic factors, and physical chronic conditions such as diabetes and obesity are associated for inpatients with SUD. This study utilized the Virginia Health Information (VHI) database. The VHI database is managed by the Virginia Department of Health and includes discharge data from public hospitals in Virginia. Inpatients 12 years and older with an SUD diagnosis and a discharge from 2018 to 2022 were included. This study was approved by the Eastern Virginia Medical School Institutional Review Board as secondary data research (IRB# 24-01-NH-0022).

### 2.2. Data Collection

To evaluate the association of mental illness comorbid status with COVID-19, chronic physical medical conditions, and demographic characteristics, the mental illness comorbid variable was operationalized as a categorical variable including “none” (patient has no mental illness diagnoses), “one” (patient has one mental illness diagnosis), or “two or more” (patient has at least two co-occurring mental illness diagnoses). We included the following mental illness diagnoses: anxiety, depression, PTSD, psychosis, suicidal ideation, insomnia, and ADHD. These have been reported in the literature to have increased from pre-COVID to post-COVID [1,10,11,12,13,14,41] and are suspected to have formed by neurotransmission dysfunction/dysregulation [14,33,43,44]. For the purposes of this study, SUD was not classified as a mental illness to differentiate from the mental illness comorbidity independent variable. Thus, comorbidity in this study refers to having at least two co-occurring mental illness diagnoses (anxiety, depression, PTSD, psychosis, suicidal ideation, insomnia, and/or ADHD). The Virginia Department of Health regularly collects alcohol or drug abuse disorder data in the VHI database; this was used to identify the SUD population. VHI data are provided by the state and contain Virginia hospital discharges as de-identified patient-level data [45]. VHI’s data dictionary can be found online at https://www.vhi.org/files_admin/PDFs_to_download_from_web/Data_Directory.pdf?a=695829 (accessed on 5 January 2026). Virginia Health Information (VHI) collects data using billing claims that adhere to the current National Uniform Billing Manual. VHI edits all data at the record level for integrity. Records with “fatal” errors in admission date, discharge date patient status at discharge, date of birth, principal diagnosis, or principal procedures are excluded from the database. Duplicate records are also excluded. Other field errors are designated as “error” or “unknown” [45]. VHI uses ICD-10 algorithms to collect diagnoses. Clinical scenarios are mapped to specific ICD-10 diagnosis codes. To ensure accuracy and specificity for conditions, algorithm logic is used such as inpatient codes within a timeframe. Healthcare providers submit claims with these ICD-10 codes. VHI uses algorithm logic detailed in the Virginia All Payers Claims Database (APCD) Data Submission Manual to validate data [46]. VHI data were provided as Excel files via Dropbox, a cloud storage service used to store, sync, and share files online. An abstraction form was not needed. Variables of interest were extracted, and a new file was created using Structured Query Language 2022 and Statistical Analysis System (SAS 9.4).

We included the sociodemographic variables available in the VHI dataset: age, race, sex, and insurance (Table 1). Other important demographic variables such as income or education were not available in the VHI dataset. The physical illnesses included in this study were selected based on literature findings about medical conditions that are common among individuals with COVID-19. Based on literature findings, cardiovascular disease (CVD), hypertension (HTN), diabetes, cancer, chronic obstructive pulmonary disease (COPD), obesity, and human immunodeficiency virus/acquired immunodeficiency syndrome (HIV/AIDS) [47,48,49] were included. ICD-10 codes in diagnosis 1 through 18 were used to identify the physical illnesses (COVID-19, Long COVID, CVD, HTN, diabetes, cancer, COPD, obesity, and HIV/AIDS) and the mental illnesses (anxiety, depression, PTSD, psychosis, suicidal ideation, insomnia, and ADHD).

The diagnosis code for COVID-19 (ICD-10 is U07.1) was introduced on 1 April 2020 [50], and the Long COVID ICD-10 code became available on 1 October 2021 [51]. Thus, the pre-COVID time in this study is from January 2018 to March 2020, while the post-COVID time is from April 2020 to December 2022. COVID-19 and Long COVID diagnoses were combined due to the low number of Long COVID cases (n = 82) and the support of the literature review indicating a relationship between mental illness and COVID-19 and between mental illness and Long COVID [1,2,16,17,18,19].

### 2.3. Statistical Analysis

Since the number of hospital visits declined from pre- to post-COVID [20,21,22,23,24,25], two-proportion Z tests were used to evaluate differences in the proportion of mental illness comorbidity from pre- to post-COVID-19 pandemic (January 2018 to December 2022). A continuity correction was not needed due to the large sample size. The associations between mental illness comorbidity, COVID-19/Long COVID, physical illness, or demographic characteristics for inpatients with SUD were evaluated using unadjusted bivariate Chi-square tests for inpatients in the post-COVID time frame only (April 2020 to December 2022) since the ICD-10 code for COVID-19 did not become available until 1 April 2020. *p*-values of less than 0.05 were considered significant. Data were analyzed using SAS 9.4.

## 3. Results

The demographic characteristics for the inpatients in the pre-COVID period were not significantly different from the inpatients in the post-COVID period. The majority of inpatients for both the pre- and post-COVID times were mostly male (pre-62.29%, post-63.20%), White (pre-64.53%, post-65.35%), between the ages of 40 and 64 years (pre-49.96%, post-48.98%), and with health insurance (pre-89.78%, post-96.40%); see Table 1.

### 3.1. Results for Research Question # 1: Are There Increases in Rates of Single Mental Illness Diagnosis or Increases in Rates of Comorbid Mental Illness Diagnoses Among Individuals with SUD After the COVID-19 Pandemic?

The proportion of SUD inpatients without mental illness significantly decreased from pre-pandemic to post-pandemic (*p* < 0.001), while the proportion of SUD inpatients with a single mental illness diagnosis (anxiety, depression, PTSD, psychosis, suicidal ideation, insomnia, ADHD) and comorbid mental illness diagnoses (two or more) significantly increased from pre-pandemic to post-pandemic (*p* < 0.001) (Table 2).

### 3.2. Results for Research Question #2: Is Mental Illness Comorbidity Associated with COVID-19/Long COVID, Chronic Physical Illness, or Demographic Characteristics Among Inpatients with an SUD Diagnosis?

Mental illness comorbid status was significantly associated with the demographic variables sex, age, race, and health insurance (*p* < 0.0001) (Table 3). SUD inpatients most likely to have two or more mental illness comorbid diagnoses included adolescents 12–19 years (30.26%), females (23.85%), American Indians (22.05%), and those who had health insurance (20.56%). These groups were also more likely than other groups to have a single mental illness diagnosis (i.e., 38.13%, 30.98%, 31.50%, respectively) (Table 3).

Mental illness comorbid status was significantly associated with the physical illness variables COVID-19/Long COVID, HIV/AIDS, obesity, COPD, CVD, cancer, diabetes, and hypertension (*p* < 0.0001) (Table 4). The majority of inpatients with single/comorbid mental illness were adolescents and young adults and had lower rates of physical chronic illnesses, with the exception of HIV/AIDS. The results indicate a higher prevalence of single/comorbid mental illness among inpatients with an HIV/AIDS diagnosis. Inpatients with uncontrolled diabetes had higher rates of single/comorbid mental illness compared to inpatients with controlled diabetes (Table 4). Overall, individuals with HIV/AIDS (59.24%) had the highest rates of single/comorbid mental illness, followed by obesity (43.97%) and uncontrolled diabetes (43.64%). Individuals with COVID-19/Long COVID (41.62%), hypertension (41.25%), controlled diabetes (37.37%), COPD (35.35%), CVD (32.76%), and cancer (30.05%) had high rates of single/comorbid mental illness (Table 4).

## 4. Discussion

The purpose of this study was to evaluate whether mental illness comorbidity increased from pre- to post-pandemic, and whether mental illness comorbid status is associated with COVID-19/Long COVID, chronic physical illness, or demographic factors among inpatients with SUD.

This study indicates a significant increase in single and comorbid mental illness from pre- to post-COVID time. This finding is significant given the bi-directional relationship between mental and physical illness. COVID-19/Long COVID may result in mental illness risk factors for SUD by altering the dopamine and serotonin synthetic pathways, leading to neurotransmission dysregulation/dysfunction [16,43,44]. In addition, people with multiple mental illnesses are more likely to experience greater combined disease severity and more likely to develop SUD [52]. According to the National Institute on Drug Abuse, people with SUD and mental illness are less likely to adhere to treatment. Substance use can also reduce the effectiveness of medications [42]. Innovative healthcare solutions may be necessary to address the rise in complex medical needs. For example, integrated models using tailored treatment plans that are coordinated by multidisciplinary teams could be implemented and evaluated for populations with SUD and mental illness.

The majority of SUD inpatients with comorbid mental illness diagnoses were adolescents who did not have a chronic physical illness such as cardiovascular disease or hypertension. This aligns with other findings suggesting these chronic conditions are typically developed after the adolescent years [53]. The increase in mental illness comorbidity among adolescents is concerning and calls for public health action. Community- and school-based screening efforts and interventions may prevent the worsening of mental and physical health outcomes for adolescents. Insured inpatients had higher rates of mental illness comorbidity compared to uninsured inpatients. This finding may be partially explained by other studies suggesting uninsured individuals with mental illness are more likely to visit emergency rooms and less likely to be hospitalized [54,55]. Alternatively, insured individuals interact with the healthcare system more frequently, leading to more opportunities for mental illnesses to be diagnosed and a perception of greater comorbidity among this group [56]. Future studies could evaluate mental illness comorbidity trends for uninsured individuals in emergency rooms and community settings. American Indians had the highest rates of comorbid and single mental illness. A similar finding suggests American Indians are more likely to experience a disproportionately higher rate of mental illness such as PTSD, depression, anxiety, and suicide [57]. Policies that promote accessible and culturally appropriate mental healthcare to this minority group are needed.

Among SUD inpatients with a chronic physical illness, HIV/AIDS had the highest rates of comorbid and single mental illness. The literature reports that individuals with HIV are at increased risk of developing mental illness [58]. In addition, a study suggests 37.6% of adults with HIV had a history of SUD [59]. Moreover, people with HIV have weaker immune systems and are more likely to experience more severe symptoms from COVID-19 [60]. This finding is significant given that individuals with HIV, a vulnerable population, may experience a greater health burden and a greater likelihood of developing SUD during a pandemic or other stressful events. Long-term integrated physical and mental healthcare services may be necessary to improve the health outcomes of people with HIV/AIDS.

This study has important limitations. People delayed seeking mental health services during the pandemic, which may have impacted the results. Seasonality could confound the results as there may have been changes in policies, bed-capacity shifts, coding, and case-mix changes. The true incidence of COVID-19 during the early pandemic period may be affected by testing shortages, rapid changes in case definitions, and large numbers of asymptomatic and mildly symptomatic cases being missed [61,62]. The Long COVID ICD-10 code (U09.09) was not introduced until October 2021 and there was differential ascertainment over time [51]. These potential confounding effects were not adjusted with statistical models. The complexity of the mental illness comorbid status is limited by the inclusion of only those mental illnesses that are reported in the literature to have increased after the COVID-19 pandemic and that are suspected to be formed by neurotransmission dysfunction/dysregulation. There are multiple mental illnesses that have not been studied in relation to COVID-19 or Long COVID. The severity of diagnoses was not available in the VHI dataset. However, there is evidence that people with comorbid mental illnesses are more likely to experience greater combined disease severity [52,63,64,65]. The VHI patient discharge data are de-identified, so we were not able to determine the independence assumption. However, this study’s large sample size makes the Chi-square test less sensitive to small correlations among observations. The impact of minor dependence is reduced by the stabilization of expected counts and improving asymptotic approximations [66]. Another limitation related to the use of discharge data is that inpatients who were hospitalized for non-mental illness reasons may have incomplete data on mental illness status. Finally, causality cannot be established as this study uses a cross-sectional design. This study presents preliminary significant associations between mental illness comorbidity and age, race, sex, and chronic physical illness. Future studies may support the validity of this study’s findings by using multinomial models including potential confounding effects.

This study also has important strengths. Most of the inpatients were males. Historically, males have been underrepresented in medical research, including mental health research. For example, a systematic review including 110 studies evaluating different interventions for depression found that men were highly underrepresented across all interventional modes [67]. This study provides an opportunity for the representation of males in mental health research. The specific association between mental illness comorbidity and physical illness and demographic factors has not been evaluated before. This study demonstrates increased rates of mental illness comorbidity after the pandemic and identified significantly higher rates of comorbidity for adolescents, females, American Indians, and people with HIV/AIDS among inpatients with SUD. Although most SUD inpatients with comorbid mental illness diagnoses were mainly adolescents and had a lower coded prevalence of chronic physical illness, older SUD inpatients (40 years and older) with COVID-19/Long COVID, obesity, uncontrolled diabetes, and hypertension had higher rates of comorbid mental illness compared to other chronic physical illnesses. Compared to previous findings addressing increased rates of single mental illness, the evaluation of comorbid mental illness prevalence adds a more comprehensive understanding of the population-level mental health burden and provides an insight into clinical complexity and treatment needs. COVID-19 vaccination, management of uncontrolled diabetes, health promotion programs, and integrated holistic clinical services may alleviate the health burden carried by individuals with mental illnesses.

## 5. Conclusions

In conclusion, the mental illness comorbidity rates before and after the pandemic and the evaluation of demographic and clinical characteristics at risk for inpatients with SUD have not been studied prior to this study. This study provides a new insight into the increased rates of comorbid mental illness after the pandemic and the significant association with age, race, sex, and chronic physical illness. In particular, SUD inpatients with HIV/AIDS were found to have the highest rates of comorbid mental illness compared to other chronic physical illnesses. This study highlights the importance of continued health promotion efforts and policies in improving the overall health of individuals. These findings are important for healthcare leaders who may need to use innovative healthcare solutions to address the increase in complex medical needs when facing rising rates of comorbid mental illnesses.

## Figures and Tables

**Table 1:** Demographics for Discharges with SUD ^a^, 2018–2022, N = 233,017.

Variables	Pre-COVID *n* (%)	Post-COVID *n* (%)
**Sex**		
Female	52,527 (37.71%)	34,488 (36.80%)
Male	86,762 (62.29%)	59,218 (63.20%)
**Race**		
American Indian	147 (0.11%)	127 (0.14%)
Asian	868 (0.63%)	503 (0.55%)
Black	42,121 (30.81%)	27,575 (29.96%)
Black (Hispanic)	98 (0.07%)	68 (0.07%)
Hispanic	1269 (0.93%)	979 (1.06%)
White	88,234 (64.53%)	60,156 (65.35%)
Other	3987 (2.92%)	2641 (2.87%)
**Age Group**		
12 to 19 years	4594 (3.30%)	2452 (2.62%)
20 to 39 years	42,693 (30.65%)	30,320 (32.35%)
40 to 64 years	69,598 (49.96%)	45,906 (48.98%)
65+ years	22,414 (16.09%)	15,040 (16.05%)
**Insurance**		
Yes	124,619 (89.78%)	89,931 (96.40%)
No	14,181 (10.22%)	3362 (3.60%)

^a^ SUD = substance use disorder.

**Table 2:** Z Tests for Proportions of Mental Illness Diagnoses from 2018 to 2022. N = 233,017.

Single vs. Comorbid Mental Illness Dx ^a^	Pre-COVID Proportion, N = 139,299	Post-COVID Proportion, N = 93,718	Z Test	*p*
None	83,386 (59.86%)	50,384 (53.76%)	29.20	*p* < 0.0001 *
1 Mental illness	32,279 (23.17%)	24,568 (26.22%)	16.80	*p* < 0.0001 *
2+ Mental illnesses	23,634 (16.97%)	18,766 (20.02%)	18.70	*p* < 0.0001 *

^a^ Dx = diagnosis, * *p* values are significant.

**Table 3:** Chi Square Test for Mental Illness Comorbid Status by Demographic Characteristic for Inpatients with SUD ^a^.

	Mental Illness Diagnosis			
Demographics	Two or More	One	None	*p* Value
**Sex**				
Male (N = 59,218)	10,538 (17.80%)	13,879 (23.44%)	34,801 (58.77%)	<0.0001 *
Female (N = 34,488)	8227 (23.85%)	10,686 (30.98%)	15,575 (45.16%)	
**Age**				
12 to 19 years (N = 2452)	742 (30.26%)	935 (38.13%)	775 (31.61%)	<0.0001 *
20 to 39 years (N = 30,320)	8029 (26.48%)	9254 (30.52%)	13,037 (43.00%)	
40 to 64 years (N = 45,906)	8590 (18.71%)	11,578 (25.22%)	25,738 (56.07%)	
65+ years (N = 15,040)	1405 (9.34%)	2801 (18.62%)	10,834 (72.03%)	
**Race**				
Black (N = 27,575)	4792 (17.38%)	6211 (22.52%)	16,572 (60.10%	<0.0001 *
White (N = 60,156)	12,926 (21.49%)	16,861 (28.03%)	30,369 (50.48%)	
America Indian (N = 127)	28 (22.05%)	40 (31.50%)	59 (46.46%)	
Asian (N = 503)	78 (15.51%)	118 (23.46%)	307 (61.03%)	
Black Hispanic (N = 68)	10 (14.71%)	16 (23.53%)	42 (61.76%)	
Hispanic (N = 979)	183 (18.69%)	236 (24.11%)	560 (57.20%)	
Other (N = 2641)	468 (17.72%)	618 (23.40%)	1555 (58.88%)	
**Insurance**				
Yes (N = 89,931)	18,224 (20.56%)	23,803 (26.47%)	47,904 (53.27%)	<0.0001 *
No (N = 3362)	465 (13.83%)	652 (19.39%)	2245 (66.78%)	

^a^ Only inpatients in Post-COVID time (April 2020 to December 2022). * *p* values are statistically significant.

**Table 4:** Chi Square Test for Mental Illness Comorbid Status by Physical Illness for Inpatients with SUD ^a^.

	Mental Illness Diagnosis			
Physical Illness	Two or More *n* (%)	One *n* (%)	None *n* (%)	*p* Value
**HIV/AIDS**				
Yes (N = 871)	239 (27.44%)	277 (31.80%)	355 (40.76%)	<0.0001 *
No (N = 92,847)	18,527 (19.95%)	24,291 (26.16%)	50,029 (53.88%)	
**Obesity**				
Yes (N = 11,486)	2043 (17.79%)	2984 (25.98%)	6459 (56.23%)	<0.0001 *
No (N = 82,232)	16,723 (20.34%)	21,584 (26.25%)	43,925 (53.42%)	
**COPD**				
Yes (N = 2732)	343 (12.55%)	623 (22.80%)	1766 (64.64%)	<0.0001 *
No (N = 90,986)	18,423 (20.25%)	23,945 (26.32%)	48,618 (53.43%)	
**CVD**				
Yes (N = 16,581)	1952 (11.77%)	3480 (20.99%)	11,149 (67.24%)	<0.0001 *
No (N = 77,137)	16,814 (21.80%)	21,088 (27.34%)	39,235 (50.86%)	
**Cancer**				
Yes (N = 2223)	232 (10.44%)	436 (19.61%)	1555 (69.95%)	<0.0001 *
No (N = 91,495)	18,534 (20.26%)	24,132 (26.38%)	48,829 (53.37%)	
**Controlled** **Diabetes ^b^**				
Yes (N = 9623)	1337 (13.89%)	2259 (23.48%)	6027 (62.63%)	<0.0001 *
No (N = 84,095)	17,429 (20.73%)	22,309 (26.53%)	44,357 (52.75%)	
**Uncontrolled** **Diabetes ^c^**				
Yes (N = 5700)	1093 (19.18%)	1394 (24.46%)	3213 (56.37%)	0.0002 *
No (N = 88,018)	17,673 (20.08%)	23,174 (26.33%)	47,171 (53.59%)	
**HTN**				
Yes (N = 41,664)	7087 (17.01%)	10,308 (24.24%)	24,269 (58.25%)	<0.0001 *
No (N = 52,054)	11,679 (22.44%)	14,260 (27.39%)	26,115 (50.17%)	
**COVID-19/** **Long COVID**				
Yes (N = 3025)	548 (18.12%)	711 (23.50%)	1766 (58.38%)	<0.0001 *
No (N = 90,693)	18,218 (20.09%)	23,857 (26.31%)	48,618 (53.61%)	

^a^ Only inpatients in Post-COVID time (April 2020 to December 2022), ^b^ A1c levels below 7%, ^c^ A1c levels above 7%, * *p* values are statistically significant.

## Data Availability

The Virginia Department of Health owns and manages the Virginia Health Information database. Data cannot be shared by the authors. Data can be purchased using the following web link: Virginia Health Information Data Products https://www.vhi.org/Products/default.asp (accessed on 5 January 2026).

## Abbreviations

The following abbreviations are used in this manuscript:

ADHD	attention deficit hyperactivity disorder
COPD	chronic obstructive pulmonary disease
CVD	cardiovascular disease
HIV/AIDS	human immunodeficiency virus/acquired immunodeficiency syndrome
MI	mental illness
PTSD	posttraumatic stress disorder
SUD	substance use disorder
